# Subculture wars: The struggle for the vape industry

**DOI:** 10.1111/1468-4446.12981

**Published:** 2022-11-03

**Authors:** Frances Thirlway

**Affiliations:** ^1^ Sociology Department University of York York UK

**Keywords:** authenticity, bourdieu, consumption, field analysis, subculture, vape industry

## Abstract

Drawing on a 2‐year study, I argue that the UK vape industry is engaged in a classificatory struggle between a subcultural industry and its “other”, the mainstream industry. I build on Thornton's analysis of club culture to characterize the subcultural vape industry as a community of taste built round a masculine aesthetic and a commitment to authenticity and DIY practice. Its attachment to complex systems and masculine spaces risked excluding customers without specialist knowledge or interest. The mainstream industry included tobacco companies which promoted vaping as a complementary category to smoking, linking their own vaping products to historic meanings of the cigarette as a lifestyle product. This task was hampered by the toxic legacy of combusted tobacco and its increasing reversion to a generic category rather than a branded product. Finally, the success of the price‐focused vaping industry has been largely overlooked, but suggests that for most consumers, electronic cigarettes are still a contrasting category to combusted tobacco and are purchased largely on price. I conclude that the exclusion of a feminized, classed “other” is a defining element of subcultural formation, itself an overwhelmingly male mechanism of group identity construction.

## 1 INTRODUCTION

Vaping began to emerge as a practice in countries of the global north after sales increased dramatically from around 2010 following the introduction of electronic cigarettes into European and North American markets in 2006 (Fagerstrom et al., 2015). Journalists quickly characterized vapers as a subculture (Couts, 2013; McGrady, 2019; Park, 2013; Sottille, 2014); a typical account described “a hardcore of early‐adopter metalheads with gauged ears and box mods” (Smith IV, 2015). Sociological research with a broader sample identified a divide between two groups of vapers, one of whom had subcultural characteristics whilst the other explicitly rejected these (Farrimond, 2017; McCausland et al., 2020; Thirlway, 2016, 2018; Tokle and Pedersen, 2019). At the same time, vaping became controversial in tobacco control circles. Some public health advocates embraced e‐cigarettes as a lifeline for smokers struggling to quit, whilst others condemned them as a new source of addiction and possible gateway into tobacco use (Green et al., 2016). Research on the vape industry itself has been limited. Studies have either analyzed the tobacco industry's market share of the vape industry (Mathers et al., 2019), quantified e‐cigarette marketing efforts (de Andrade et al., 2013; Haardörfer et al., 2017) or focused on retail outlets (Hammond et al., 2015), particularly specialist vape shops (Pattinson et al., 2018; Sussman and Barker, 2016). Only a handful of studies from the US have provided a broad overview of the vape market as a whole (Levy et al., 2019, 2021, 2022).

In this article, I draw on data from a 2‐year study in the UK to argue that the vape market is characterized by a classificatory struggle between a subcultural vape industry and its “other”, the mainstream vape industry. I situate my study within the literature on symbolic boundaries and classificatory struggles, arguing that the vape market constitutes a field of relational forces (Bourdieu, 1983; Fligstein, 2018). The sociological analysis of symbolic boundaries (Pachucki et al., 2007) goes back both to Durkheim's work on classification systems and their relationship with the moral order and to Weber's emphasis on their role in the creation of inequality (Lamont et al., 2001). Bourdieu's work on classification struggles and cultural production led to a particular focus on how boundaries intersect with the production of inequalities (Bourdieu, 1984; Bourdieu and Passeron, 2018 [1970], cited in Lamont et al., 2001), 2018 [1970]. Boundary work has been explored in other research, however, including the construction of consumer product categories such as food and tobacco (Carroll and Swaminathan, 2000; Rao et al., 2003). In relation to vape products, a recent study has suggested that attempts by early e‐cigarette entrepreneurs to position their product as a contrasting category to cigarettes were impeded by US regulators framing vapes as tobacco products and by the tobacco industry entering the vape industry, transferring stigma from the existing smoking category to vaping as a new practice (Hsu and Grodal, 2021).

### 1.1 Subculture and the mainstream

Sociologists of the Chicago School in the 1920s first distinguished “deviant” groups from the “mainstream”, and Merton linked the formation of subcultures with the rejection of mainstream values (Merton, 2017 [1938], cited in Williams, 2007). However, probably the most influential work emerged at the Birmingham Centre for Contemporary Cultural Studies (CCCS), focusing on the oppositional strategies of youth subcultures as read in their clothing and music styles (Hall and Jefferson, 1993; Hebdige, 1979). Although early critiques took issue with the CCCS's naturalization of subculture as masculine (McRobbie and Garber, 1993), studies of music or style subcultures continued to be largely celebratory until Thornton's landmark study of club culture showed that the imagined mainstream against which subcultures constructed themselves was implicitly working‐class and female ('Sharon and Tracy dance around their handbags', Thornton, 1996, p. 99). Later critics set out how music subcultures privilege connoisseurship against “mass” taste, with the (male) subculture fan constructed as a “collector” as opposed to the feminized, supposedly passive shopper or consumer (Hollows, 2003); and how specific material culture ‐ record collections for example, ‐ provides the raw material around which rituals of male interaction take place (Straw, 1997). Beyond music studies, Nagle's research into misogyny on the anonymous online forum 4chan exposed an alt‐right culture which railed against “Chad and Stacy” “normies”, claimed there were “no girls” on the authentic Internet (Nagle, 2017, p. 108) and blamed female vanity for shaping mainstream social media around “selfies”.

Whilst CCCS scholars took the mainstream for granted (Huber, 2007, 2013) as the mass culture against which authentic subculture rebelled (Williams, 2007), Thornton found the mainstream was imagined rather than experienced by the clubbers she studied. She argued that academic accounts accepted the authentic/mainstream dichotomy uncritically, and that so‐called authentic culture remained the prerogative of men, whilst women were identified with “mass” culture (Huyssen, 1986). This was not so much because women or girls' sociality played out in different areas (McRobbie and Garber, 1993) but because they were less sectarian about music taste, refusing to engage in subcultural distinction as a competitive sport (Thornton, 1996, p. 104). The idea of the authentic also featured in the 1970s punk rejection of mass‐produced culture in favour of consumers becoming producers by making their own, “Do It Yourself” (DIY) culture (Duncombe, 1997, cited in Beaver, 2012). Authenticity was favourably contrasted with “selling out” or allowing DIY culture to be commercialized and ultimately subsumed into the mainstream. More recent studies of craft beer production and consumption have also focused uncritically on authenticity and the DIY ethos (Ocejo, 2017; Thurnell‐Read, 2019). Others have been more mindful of classificatory struggles and boundary work, for instance in relation to the whiteness of craft beer cultures (Chapman and Brunsma, 2020), its conflation of authenticity and masculinity (Darwin, 2018) and the entanglement of craft brewing with urban gentrification processes (Wallace, 2019).

## 2 METHODS

This article builds on ethnographic and qualitative research with smokers and vapers in the North of England from 2012 to 2022; fieldwork for this specific project took place between 2017 and 2019. Funded by Cancer Research UK, the study involved interviews with smokers and quitters in deprived areas to explore whether vaping could help them stop smoking (Thirlway, 2019), plus observations and interviews in specialist vape shops and other vape sales locations. UK policy makers and public health officials have generally been supportive of vaping's potential to address health inequalities by reducing smoking in deprived groups, making the UK a keenly‐watched natural experiment (Berridge et al., 2020; Green et al., 2016). The UK is the second‐largest vape market after the US, with an estimated 3.6 million vapers (ASH, 2021) and a market worth one to two billion pounds per annum (Brown, 2020).

Fieldwork with smokers, vapers and vape shops took place in two contrasting deprived areas: an urban neighbourhood close to a big city centre, and a small town and its semi‐rural surroundings. I visited shops in a wider radius, reflecting consumers' buying habits beyond their immediate neighbourhood. Some shops were located in deprived areas, but others were in high‐footfall mixed areas for example, in shopping centres, close to railway stations or on popular bus routes or road arteries into the city. I spoke to staff in 28 shops, including branches of the three biggest independent vape companies. I introduced myself to staff and explained what I was doing; where agreed, I recorded an audio‐interview of up to 60 min. Ethical approval for the research was obtained from the Economics, Law, Management, Politics and Sociology (ELMPS) Ethics Committee at the University of York. I talked research participants through an information sheet which explained the purpose of my study, the identity of the funders, anticipated use of data and other issues before obtaining written consent (ASA, 2011). I have obscured research locations and changed names and other details of participants in the text to protect their anonymity. Research on what I will call the mainstream vape industry took a different route since its products are mainly sold through non‐specialist retailers. I kept note of the vaping products available in convenience stores, garage forecourts, supermarkets and discount stores, but conversations in these outlets were limited since staff had little knowledge of the vape products they sold. I read market reports on the tobacco and vaping industries, followed vaping industry and convenience store news sites and blogs, subscribed to vape and tobacco industry job alerts and consulted business accounts filed with Companies House.

### 2.1 The global vape industry

Vaping involves heating up propylene glycol or vegetable glycerine (PG/VG), nicotine and flavours until this “e‐liquid” turns into vapour which can be inhaled. Electronic cigarettes were invented in China in 2003 and 90 per cent of vaping hardware is still made in Shenzhen. Whilst the first e‐liquids were also made in China, other countries including the US and UK now have a significant e‐liquid industry catering to local tastes. Product distribution takes place either in supermarkets, convenience stores and discount shops or through thousands of specialist vape shops on the high street and to a lesser extent online. Most of these are small or micro‐businesses with often just one and rarely more than five premises, reflecting the low market concentration of the vape industry in both the UK and the US (Anastasopoulou, 2019; Levy et al., 2019). Significant industry players in the UK include 2010s vape start‐ups as well as brands bought up by a diversifying tobacco industry or by other industries with strong distribution networks. Top UK companies include independents Totally Wicked, Flavour Warehouse and Supreme Imports' vape subsidiary 88vape. Tobacco industry vape subsidiaries include BAT's Vype/Vuse, Imperial Brands' Blu/Myblu and Japan Tobacco International's Logic brand.

Most vapers in the UK buy bottles of e‐liquid to refill their “open” device, which might be pen‐shaped, a smaller pod device or a larger box mod (West et al., 2021). Alternatively, vapers can buy ready‐filled proprietary closed cartridges for their pod vape (Hammond et al., 2021) or closed disposable vapes (Leventhal et al., 2021). Because devices with higher power have generally provided a better experience, the size of popular vapes has increased over time to accommodate bigger batteries. This changed in 2019 with the advent of nicotine salts, a new configuration of nicotine which can provide a satisfying experience even with a smaller battery. Nicotine salts facilitated the rise of small pod devices such as Juul, followed by disposables such as the Elf Bar and Geek Bar in the UK and Puff Bar in the US.

### 2.2 Findings

#### 2.2.1 The subcultural vape industry

In her study of club culture, Thornton identified subcultural characteristics including a shared material culture, a preoccupation with authenticity, the blending of consumers and producers, male sociality, and most critically, self‐definition against a mainstream “other” (Thornton, 1996). I recognized these in the vape industry and I explore them here. I begin with shared material culture. John has a luxuriant beard, ear stretchers and tattoo “sleeves”. His shop decor expresses his love of “*all things skater*, *biker and tattoos*”. As well as vaping supplies, John sells motorcycle apparel and parts and has an in‐store “*neo‐traditional barbershop*”. The black‐painted shop sits just outside the city centre and on the gentrification frontline; construction workers erecting the nearby flats eat their sandwiches with John's excellent coffee. A few miles down the road, Ryan works in a vape concession in a newsagent's shop. He too has a beard, ear stretchers and a trucker cap and used to play in a hardcore punk rock band sponsored by an energy drinks brand; he was attracted by vaping's “*tattoos and beards and caps*” image, he tells me. Besides, “*where else would I get a job?*” he says, pointing to his neck tattoo. A hundred miles away, Mike plays games on his phone between customers, and decorates his shop with skulls and fantasy paraphernalia. He has a large beard, a short mohawk haircut and a mournful expression. According to the label on the fuel cap, Mike's car “*runs on zombie dust*”. During Covid‐19 pandemic‐related restrictions, Mike used a novelty skeleton arm to pass his card machine out to customers outside. Most of these are male; it's a “*men and gadgets*” thing, he says. Of his few women customers, half like “*dinky*, *discreet*” vapes, the other half are “*sub‐ohming*,^1^ *with tattoos and piercings*”.

Although the three men I have described had different interests, they were part of the same “taste culture” (Thornton, 1996, p. 3) with links to groups such as “*niche tattoo culture*, *urban skating communities or motorcycle detailing shops*” (Smith IV, 2015). Vaping hobbyists were more visible in the shops than ordinary ex‐smokers, coming in more often and staying for longer. In theory, businesses recognized the need to avoid “*the dark*, *grungy tattoo parlour look*” (Liam, large vape chain) “*with weird posters on the wall*” (Josh, one shop), but some of the biggest UK vape companies have a cartoon character logo, and the brand imagery for many e‐liquids still suggests masculine, heavy metal or fantasy themes. One UK company uses a clenched fist logo to enjoin customers to “*never back down*”; its bullet‐shaped e‐liquid bottles make “*bold statements of non‐conformity… created to secure mass disruption*”. The company's creative director (and sometime session drummer) 2021 online profile shows him wearing a trucker cap, a beard and ear stretchers. Its 2016 Vape Expo prize‐winning stand “*included a bold*, *branded SWAT style van… visitors were taken captive and dressed in prisoner boiler suits before being bundled into the interrogation chamber*” (Milton Keynes Citizen, 2016). This aesthetic could be intimidating to some customers; support worker Caitlin (30) first met vape shop staff at a public engagement event I organized in 2017, and was surprised by how approachable they were. She had never dared go into a vape shop: “*I thought I would have to know what to ask for*”, she said.

As well as sharing a particular aesthetic, large parts of the vape industry had a typically subcultural preoccupation with preserving authenticity and the DIY ethos. Early adopter Rob complained that shops were no longer run by enthusiasts but by “*people who just want to make money*”. Specialist vape retailers disliked tobacco‐owned vape brands partly because they suspected the tobacco industry wanted to kill them off or take them over, but they also objected to tobacco industry representatives' professionalized image, bringing “*too many suits*” into vape shows. The DIY ethos was apparent from the fact that even small shops made and sold their own liquids: shop fitter Josh started making liquids for himself; friends and family liked them and he built up a business online and later opened a high street shop. Like Josh, factory worker Mike switched from smoking to vaping; eventually his local vape shop partnered him to start his own business and supplied him with merchandise, including their own line of e‐liquids.

There was considerable overlap between consumers and producers (Thornton, 1996, p. 26). Almost all shop staff were former smokers who vaped themselves, with many job advertisements making one or 2 years vaping a requirement. Many shops had a small group of “customers‐plus”: young men in their twenties or early thirties who hung around, discussed kit, helped out behind the counter and often ended up as staff themselves. The black vinyl sofa found in many shops signaled that this was not just a transactional space, but somewhere where enthusiasts might gather. Vape businesses which were less keen on what Mike called his “*shop rats*” had to work hard to discourage groups of young men from hanging around the shop and vaping (Liam, large vape chain employee). Ciaran (owner, one shop) complained that “*I get a lot of hobbyists in here as well*, *so it could put people off with the amount of vapour that some people produce*”.

As in Thornton's analysis, this sociality was almost entirely male. Only a quarter of the 50 or so vape shop staff and owners I met were women, and the vape industry magazines which lay around the shops rarely profiled any women in the vape business. Of the women shop staff I met, a handful could be characterized alongside male colleagues as possessing subcultural capital (Thornton, 1996) in the shape of biker/metal‐style clothing, interests in gaming or fantasy, extensive technical knowledge and ownership of impressive collections of vaping devices. Others had joined a business set up by a male relative; they showed less interest in the technical aspects of vaping devices than men such as James (employee, chain of four shops), who said “*I love technology more than people*, *to be honest*”. Chloe, a former hairdresser who now worked in her brother‐in‐law's vape business (three shops), told me that her male colleague: “*knows people by their kit*”. In contrast, “*I know their lives*”, she said. Geoff (owner of one shop, plus online business and own line of e‐liquids) recognized the industry was off‐putting to many women; his wife visited a vape show with him but refused to return because the only women there were the models paid to attract customers to the stands; this matched my own observations at a vape show in 2017. One vape shop owner said he would have liked to employ more women but that it was hard to recruit women vapers because they “*could make more money posting videos of themselves vaping on social media*” (Josh, internet sales plus one shop), echoing 4chan users' misogynist complaints that women using the internet were vain and obsessed with “selfies” (Nagle, 2017). Popular e‐liquid names reproduced male locker‐room culture or geeky in‐jokes. “Heisenberg” referenced the TV show “Breaking Bad” drug dealer's physicist pseudonym (Hedash, 2020); “Milf's Milk”, illustrated with a cartoon of a busty woman breastfeeding an infant, was based on an acronym used by young men to describe attractive older women.

The final subcultural aspect of the specialist vape industry and a key part of my argument was its self‐definition in opposition to an imagined mainstream industry. This included two contrasting categories which I characterize as “lifestyle” and “generic” vaping, and explore in more detail in the next section. Lifestyle vaping involved products whose selling point was that they were small, discreet and convenient. These products typically used closed cartridges in a limited range of flavours and strengths, rather than the user refilling them with a liquid of their choice. Vape shop staff disliked this removal of control as well as the fact that cartridges were expensive and their nicotine delivery was inefficient. The complexity of open systems, with messy refills and parts to change, was a small price to pay or in some cases an added attraction for a demographic which enjoyed the technical aspects of their vapes. Vape shop staff were generally contemptuous of the closed product category and by extension, of their users, who were seen as lacking commitment: they had failed to do their research and therefore lacked insider knowledge. *‘I suppose it's good for the masses'*, Ciaran (owner, one shop) told me. These “mass” users were often characterized as women and older people, and the products they favoured, which were generally smaller than the powerful devices preferred by industry insiders, were seen as feminized products. They were for “*people with pockets in their shirts*”, one vape shop employee told me, with a hint of class contempt for office workers. The assumption that smaller devices could not deliver a satisfying experience meant that the subcultural vape industry was slow to adopt the smaller “pod mods” using nicotine salts. I first saw these in 2017 in a city pawnshop where a member of staff ran vape products as a sideline, including French pod brand Bo. John (owner, two shops) used US pod brand Phix himself in September 2017, at which time he correctly predicted pod mods with nicotine salts as “*the future of vaping*”. However, most small vape shops I visited at that time were unaware of the new pod mods. It wasn't until 2019 when wholesalers were promoting refillable pods such as the Aspire Breeze, the Uwell Caliburn and the Smok Nord that these became part of their standard product range. Similarly, many specialist vape shops initially resisted the new disposables which rose sharply in popularity from 2021, partly because of an instinctive dislike of closed systems and small devices, and partly for environmental reasons as well as concerns that disposables were attracting young recreational users including a few people who had never smoked.

The other vaping category condemned by subculturalists as “mainstream” was the use of cheap, generic devices and liquids bought on market stalls or in discount shops. Like many vape shop owners, Mike refused to sell “*cissy sticks*”, as he called basic vape pens. Asked to explain, he said: *‘it's only cissies that have those little ones*… *they're more for the old fellas'*. Other shops in the poorest areas did stock cheap vape pens, arguing that if people were going to buy one it might as well be from them, but advising customers to upgrade to a £20 kit for a better experience (Gemma, family business, one shop). Connor (employee, two kiosks) sold £10 starter kits including e‐liquids, mainly to *‘old dears’*. Basic vape pens were seen as unsatisfying, poor quality items which undercut better quality brands; users were seen as lacking discernment and taste, and like the users of lifestyle products they were characterized as women or older users, but this time working‐rather than middle‐class.

#### 2.2.2 The mainstream vape industry

Thornton is critical not only of dance culture's contempt for an imagined mainstream, but also of subculture scholars such as Hebdige's uncritical acceptance of an authentic/mainstream dichotomy (Thornton, 1996, p. 93). In this second section, I supplement analysis of the subcultural industry with an additional focus on its “other”, the mainstream vape industry (Baker et al., 2013). I start with the tobacco‐owned vape industry and its strategies for dominance in the vape market. The first of these was take‐over: tobacco companies used their enormous financial resources to buy up the early “cigalike” vape companies and largely stayed with this category even after most consumers had moved to open, second‐generation systems, leading to speculation that tobacco companies were deliberately selling poor quality vaping devices to keep people smoking (Thirlway, 2015b). Shop staff interviewed in 2017 often mentioned visits from tobacco industry representatives looking to buy their businesses. As well as taking over and rebranding other companies (for instance JTI's take‐over of E‐lites, rebranded as Logic, and Imperial Brands' take‐over of My Von Erl, rebranded as MyBlu), tobacco companies also designed vape products in‐house, but struggled to achieve dominance in the market (Levy et al., 2022), and product changes were frequent. This caused confusion amongst customers: the BAT “Pebble” was launched and widely advertised on city bus stops, then discontinued within months. Both the BAT Vype/Vuse and Imperial's Blu/MyBlu were redesigned several times, and customers would come into vape shops looking for discontinued versions. BAT bought popular UK independent brand VIP in 2017, but later lost significant customer goodwill by subsuming it into another label and discontinuing original VIP flavours (VIP, 2022). In contrast, whilst new products constantly appeared in specialist shops, there was little change over time in the best‐selling “starter” open devices; older products generally continued to be available and parts such as coils were compatible across different brands and product versions.

The tobacco industry's enormous financial resources allowed it to establish a presence in the market first through massive investment and then by using its established distribution networks to get products into shops. Field sales teams promoted their vape products alongside their tobacco brands in convenience stores and supermarkets, ensuring their dominance in that sector. Vype and Blu also intermittently targeted specialist vape shops with free stock and other incentives, but specialist shop staff saw their products as overpriced and underpowered, and sales representatives' visits were erratic, reflecting parent tobacco companies' fluctuating priorities. When I started fieldwork in 2017, branded Blu display stands in vape shops were empty, but after Myblu was launched in 2019 (Convenience Store, 2019), vape shops were offered free products in return for displaying branded stickers, counter mats and posters. Similarly, vape shops saw a sudden burst of activity from Vype when it relaunched as Vuse in 2021 and distributed stock display units as well as offering a rewards system for successful sales. Over time, however, and in the absence of regular sales rep visits, the display units provided by tobacco companies tended to move to obscure corners of independent vape shops and of many corner shops too.

It was difficult for tobacco companies to offer vape products as an oppositional category to tobacco, since that would entail a recognition of the lethal nature of their main product. They tended therefore to market vape products as complementary to combusted tobacco—another way of “enjoying nicotine”, but also of maintaining smoking, allowing smokers to vape where they couldn't smoke (Tobacco Tactics, 2021). Just as they always had for tobacco products, tobacco companies used aspirational advertising and sponsorship to promote their vape brands (Hoek and Freeman, 2019; Jackler et al., 2019). An Imperial campaign showed stylish silhouettes in sunglasses asking: “*we blu do you?*” (Convenience Store, 2019), whilst BAT gave their vape products away in city centres, outside university freshers' fairs and in nightclubs (Doward, 2020) and paid models and other influencers to attend Formula 1 racing events and promote their vape products on social media (STOP, 2021). Ironically, whilst aiming their vape products at young adults and avoiding any reference to smoking cessation, tobacco industry‐owned vape brands also positioned themselves as the responsible and safe choice, emphasizing ingredient analysis, laboratory testing and point‐of‐sale age checks. They set up trade organizations to influence regulatory regimes, campaigned against “shortfills” (larger bottles of e‐liquid which are sold separately from nicotine “shots” to circumvent volume limit regulations) and inveighed against “illicit” and “dangerous” disposable devices.

The final element of the tobacco industry's strategy for market dominance was a business model which tied consumers into their brand. This started with an early interest in cigalikes and continued with the turn to pod systems using proprietary closed cartridges (de Andrade et al., 2018), which are more profitable than open devices (Levy et al., 2022). Independent vape shops complained that even when tobacco companies did sell open vape systems, they avoided compatibility with other device components for this reason. James (employee, chain of four shops) said: “*We get people come in*: “*I bought this Blu pen from [supermarket] and it doesn't work*, *and [the staff there]'ve got no idea*”… *because of course they won't*, *they're just cashiers*… *or he'll go* “*oh*, *I've already got a kit*, *can I just buy a replacement tank?*” *We'll go yeah*, *and he'll show us a Blu kit and it's like*, *no*… *you have to buy a Blu tank because the battery threads are a different size to everybody else's so you have to buy all new kit*.” The tobacco industry makes extraordinarily high profits on combusted tobacco products (Branston and Gilmore, 2015; Gilmore et al., 2010), and their preference for profitable closed systems is linked to the requirement to provide good returns to shareholders (de Andrade et al., 2018). Despite parent tobacco companies subsidies for vape subsidiaries, however, these failed to achieve market domination or indeed to break even.

I turn finally to the cheap vape product category, which the subcultural industry characterized as “mainstream” alongside the tobacco industry‐owned products discussed in the previous section. I describe this category as “generic” since products lacked a distinct brand image and were seen as interchangeable (Thirlway, 2016, 2019), so that consumers simply bought the cheapest version. Specialist vape retailers disliked the generic, discounted vape industry because they couldn't compete with them on price and felt cheaper vaping devices were less effective and cheap liquids had an unpleasant taste. Clive, who ran his own vape market stall in a deprived city neighbourhood, complained that he couldn't match the local discount store price of £1 for a 10 ml bottle of e‐liquid, as this was the same price he paid his wholesaler. He relied on customers preferring the wider range of flavours and better taste of his liquids, as well as the personal service and expertise he provided.

Like the tobacco industry‐owned brands, the generic vape industry relied on widespread, non‐specialist distribution to sell its products. Unlike the tobacco industry, however, its focus was on basic functionality at the lowest possible price. From around 2014, wholesalers were supplying corner shops and market stalls in my fieldwork areas with £5 eGo “vape pens” and counter‐top display units for many different e‐liquid brands, generally at similar prices to specialist shops, that is, 10 ml of e‐liquid for £3 to £5. However, just as tobacco companies diversified into vape products, so did many large manufacturers and distributors of other consumer goods. Top‐selling UK vape brands were produced by Supreme Imports, who started off selling batteries and light bulbs to convenience stores (88Vape and KiK vape brands), Bull Brand (smoking accessories, then Cloud 9 and K Liquid vape brands), Jucce/NVee (mobile sim cards then vapes), and DSL/Multivape (promotional products for filling stations, then vapes). Of these, it was Supreme and Bull Brand who pursued a policy of volume sales to discount shops and priced their products accordingly. By 2022, Supreme had taken over two other major independent vape companies and were probably the single biggest UK vape manufacturer and supplier.

These diversifying companies took a different approach from the tobacco‐industry owned vape subsidiaries not only by competing on price, but also in their acknowledgment that customers vape to stop smoking. Supreme Imports focus on affordability and their 88vape website includes blog pieces with tips on smoking cessation (88vape, 2022). Their advertisements on buses carried a simple message: “*Premium* (signifying quality) *e‐liquid UK‐made* (signifying safety), *only £1 per 10 ml bottle* (signifying value)”; the primary colours used (red and yellow) suggested economical rather than aspirational (Supreme Imports, 2020).

## 3 DISCUSSION AND CONCLUSIONS

I have built on Thornton's analysis of subcultural practice to characterize the UK vape industry in terms of a struggle between the subcultural and mainstream. Like spectacular youth cultures (Hebdige, 1979), subcultural vaping imposes itself on our notice and attracts media attention, but Thornton suggests that subcultures are constructed against a classed and gendered “other”. Descriptive “insider” studies of spectacular subcultures run the risk of becoming merely decorative sociology; a more critical analysis can illuminate power relations (Rojek and Turner, 2000). Although the vape industry as a field might seem far removed from club culture, I found similar processes of subculture formation to those described by Thornton. Like her, I conclude that the exclusion of a feminized, classed “other” is not so much an incidental, but a defining element of subcultural formation, itself an overwhelmingly male mechanism of group identity formation. The study of subcultures must therefore always be critical rather than celebratory.

Thornton also takes cultural studies scholars to task for their unthinking acceptance of an authentic/mainstream dichotomy. I suggest that her argument can be applied more broadly within the sociology of consumption, including cultural consumption and indeed the sociology of music. Thornton argues that older clubbers' involvement with youth culture acts as a buffer against social ageing (p. 103), but she also widens her critique to question the nature of scholarly engagement with fashionable, “cool” consumption cultures, which can appear to serve scholars' own identity formation rather than any critical analysis. As Haynes and Nowak argue, “we were never cool” (Beer, 2009; Haynes and Nowak, 2021), nor should buttressing our “cool” identity be a function of scholarship. Positionality is key here: women scholars who struggle to access subcultural registers of coolness and insider status are more likely to notice the exclusion of women from these fields (Richards and Milestone, 2000). My own critique of the subcultural vaping industry as exclusionary might not have arisen had I been either a vaper or indeed a man.

In relation to the wider literature on tobacco, sociology's weakness for spectacular subcultures may explain why the sociological literature on tobacco consumption practices is so thin. Smoking in the global north transitioned long ago from an unremarkable mass practice to a class marker and focus for middle‐class disgust (Marron, 2016; Thirlway, 2015a, 2018). Studies of differentiation within the field have found that smokers adopt defensive repertoires to distinguish their own continued smoking from moralized understandings of tobacco use, with little room for the construction of positive, never mind spectacular shared identities (Holdsworth and Robinson, 2008; Katainen, 2010; Scheffels, 2009). The stigmatized smoking and drinking practices of working‐class consumers fail to hold the attention of sociologists preoccupied with niche middle‐class consumption practices and oblivious to the cynical processes of class distinction and class contempt underlying their purported “authenticity”.

Moving beyond individual subject positions, I suggest finally that Bourdieu's work provides a methodological solution for the problem of uncritical consideration of particular subcultures. Field analysis (Bourdieu, 1983; Fligstein, 2018) requires careful attention to defining the wider field which contains the area of study. What, Bourdieu asks, constitutes the field of the art world, French higher education or in this case, the vape industry? Huber's claim that the “mainstream” is neglected and my own argument that too often we see only a part of the field, and in doing so, miss the classificatory struggle at its root, can be resolved by addressing the definitional problem of field at the start of any study. Once identified, investigation of the field may require different methodologies across its constituent sub‐fields. In the case of the vape industry, whilst the subcultural industry can be understood through engagement with its public face on the high street, a full understanding of the place of the mainstream industry may require a more strictly economic analysis (Levy et al., 2022).

Turning back to the specifics of my study, I found that the subcultural vape industry constructed its own products and practices largely as a contrasting category to mainstream vaping, that is, closed cartridge products largely promoted by the tobacco industry and to a lesser extent, cheap products. The status of vape products as an oppositional category to tobacco was taken for granted, with more energy devoted to opposing the “wrong” kind of vape products. The enthusiasm and expertise of the subcultural industry have driven the industry forward, creating a proliferation of micro‐businesses on the high street, offering product flavours to suit every palate and forcing the tobacco industry to take vaping seriously. However, the subcultural industry has sometimes been blind to the ways in which attachment to complex systems and masculine spaces excluded customers without specialist knowledge or interest, and many vape businesses were caught unawares by the new nicotine salt devices and disposables.

The tobacco industry used its spending power to buy up promising new products, relied on its extensive distribution networks to distribute these and tied customers into its products through proprietary refill cartridges. Tobacco companies were looking for a vaping device that was easy to use, but their slowness to innovate meant it was independent brands which introduced nicotine salts, first to pod mods, then to disposables. Meanwhile, the overlooked generic vape industry focused on volume distribution and basic functionality at a low price, offering a product that was effectively commoditized, that is, bought on price rather than brand (Reimann et al., 2010). This resulted from the increasing commoditization of tobacco itself: smoking is more and more concentrated among the poorest who increasingly choose the cheapest forms and brands of tobacco, with little regard to brand (Gilmore et al., 2015; Hiscock et al., 2018; Partos et al., 2017). In response, tobacco companies created super‐economy brands to keep their market share amongst poor smokers (Gilmore et al., 2013; Partos et al., 2020). The denormalization of smoking, plain packaging regulations and the ban on advertising have also eroded brand loyalty (Ford et al., 2012; Jarman, 2013), and as smokers feel more conflicted about their habit, they buy tobacco as cheaply as possible to minimize their moral exposure. Many who switch to vaping buy cheap vaping devices and e‐liquids, partly because they feel guilty about their continuing nicotine addiction (Thirlway, 2019). In addition, many users reject both the subcultural and the lifestyle category, and instead see vaping products as functional smoking cessation products (Farrimond, 2017; Thirlway, 2016; Tokle and Pedersen, 2019). For this group, vaping fits into a similar category to nicotine replacement therapy (NRT) medications, which users do not expect to enjoy; they are primarily interested in the product's effectiveness as a cessation aid (Thirlway, 2019).

Vaping continues to evolve as a disputed cultural category, and the classificatory struggle over its symbolic meaning involves contrasting players within the industry as well as consumers themselves. Studies with vapers have shown that they typically have strong views about the symbolic meaning of their own practice, whether this involves embracing or rejecting a vaper “identity” (Farrimond, 2017). Subcultural vaping holds meaning for one section of the industry and the vaping public. The tobacco industry has attempted to establish a credible lifestyle vape but has arguably lost this battle to the new disposables, although their appeal to young people is controversial. The success of the price‐focused vaping industry has been largely overlooked but suggests that for many users, electronic cigarettes are still a contrasting category to combusted tobacco, and are chosen largely on price (Pesko et al., 2019; Stoklosa et al., 2016).

## CONFLICT OF INTEREST

The author declares that there is no conflict of interest that could be perceived as prejudicing the impartiality of the research reported.

## Data Availability

Research data, that is, the author’s research notes are not shared as they form the basis of ongoing analysis. Commercially sensitive data are not shared. Some anonymized interview data that underpin the findings will be available from the data repository at the University of York following the completion of further related work. Please contact the author with any data queries.

## ENDNOTE

^1^
Sub‐ohming refers to producing large clouds of vapour by using e‐liquid with a relatively high proportion of vegetable glycerine and a vaping device with a relatively high wattage to increase flavour and vapour production.
